# RNA-Seq, physiological, and biochemical analysis of burley tobacco response to nitrogen deficiency

**DOI:** 10.1038/s41598-021-93363-w

**Published:** 2021-08-19

**Authors:** Yafei Li, Dong Chang, Xiang Zhang, Hongzhi Shi, Huijuan Yang

**Affiliations:** 1 Institute of Plant Nutrient, Resources and Environment, Henan Academy of Agricultural Sciences, Zhengzhou, 450002 China; 2 National Tobacco Cultivation & Physiology & Biochemistry Research Center, Henan Agricultural University, Zhengzhou, 450002 China; 3 Pingdingshan Branch of Henan Provincial Tobacco Company, Pingdingshan, 467002 China

**Keywords:** Biological techniques, Biotechnology, Molecular biology, Plant sciences

## Abstract

To explore the effects of nitrogen deficiency in burley tobacco, two varieties were cultivated and subjected to conditions of sufficient and deficient nitrogen. The natural characteristics of varieties TN90 and TN86 during tobacco cultivation were similar for nitrogen metabolism. Both carbon and nitrogen metabolism were significantly affected by reducing amounts of applied nitrogen. Under nitrogen-deficient conditions, average leaf biomass, root weight, photosynthetic rate (Pn), pigment levels, total nitrogen, and nitrate content of TN86 and TN90 were significantly decreased by 52.88%, 69.19%, 22.65%, 46.80%, 37.42%, and 79.15%, respectively (*p* < 0.01). Nicotine and soluble reducing sugar contents were significantly decreased by 96.67% and 95.12%, respectively, in TN86 roots (*p* < 0.01), which was consistent with the reductions in root surf area, average diameter, and root volume. Nitrogen deficiency induced 6318 differentially expressed genes in both TN90 and TN86, which were highly expressed. In total, 428 upregulated genes were analysed and found to be mainly enriched in the MAPK signalling pathway, sesquiterpenoid and triterpenoid biosynthesis, and arginine and proline metabolism. Meanwhile, 213 downregulated genes were analysed and found to be mainly enriched in photosynthesis, nitrogen metabolism, and amino acid biosynthesis. Reduced pigment content and Pn may result in low carbohydrate formation and decreased leaf biomass in burley tobacco under nitrogen-deficient conditions.

## Introduction

Burley tobacco is a well-known yellow-green leaf tobacco with low chlorophyll content and carbohydrate that accumulates in its leaves^1,2^. Mutations at the *Yellow Burley 1* (*YB1)* and *Yellow Burley 2* (*YB2*) loci have been reported, resulting in a double homozygous recessive genotype and chlorophyll deficiency. The changes may also result in low efficiency of nitrogen use and the accumulation of high levels of nitrate, which can increase tobacco-specific nitrosamines (TSNAs) in the tobacco^3,4^.

Nitrogen is a micromineral in plants and is essential for plant development and yield^5^. Nitrogen is absorbed in the roots and transported to the stem and leaves where it used as a carbon and energy source in the biosynthesis of amino acids and proteins in the cells^6^. To obtain high crop yield and increase revenue, farmers use excessive nitrogen fertiliser in tobacco cultivation^7^. Nitrogen fertiliser is applied approximately 3–5 times higher on burley tobacco than on flue-cured tobacco, but the biomass between them is similar^8,9^. Overuse of nitrogen fertiliser in tobacco cultivation may lead to environmental problems as a result of leaching and can increase nitrate accumulation in the plant tissues^10^, which negatively affects the quality of burley tobacco^3^. Nitrate levels have been found to be dozens to several hundred times greater in burley tobacco compared to that in other tobacco types^11^. The response of burley tobacco to reducing application levels of nitrogen is not completely clear. Burley tobacco varieties TN86 and TN90 are the most commonly cultivated tobacco cultivars in China, and their characteristics regarding nitrogen fertiliser application, yield, and value are similar ^12^, but their responses to conditions of nitrogen deficiency at the transcriptome level are unclear.

Nitrogen fertilisation has been reported to significantly influence plants growth traits^13^ and reducing the application of nitrogen fertiliser can effectively improve the efficiency of nitrogen use and reduce nitrogen losses^14^. Nitrogen fertilisation increases the leaf area index, leaf area duration, and leaf biomass accumulation of tobacco, while lower application of nitrogen significantly decreases nicotine, alkaloid content, and root density^15–17^. Furthermore, nitrogen deficiency has been reported to significantly affect carbon and nitrogen metabolism in crops, including rice, maize, and wheat^18–20^. However, there are currently few reports on the effects of nitrogen deficiency in burley tobacco at the transcriptome level (Supplementary Information 1).

In the current study, we performed RNA sequencing (RNA-Seq) based comparative transcriptome analysis of TN90 and TN86 grown under nitrogen-sufficient (N-sufficient) and nitrogen-deficient (N-deficient) conditions to gain an understanding of the pathways modulating carbon and nitrogen metabolism at low nitrogen levels. Pigment levels, photosynthesis rates (Pn), amounts of carbon (C) and nitrogen (N) compounds, activity of nitrogen-assimilating enzymes, and nicotine concentrations in tobacco leaves were also determined to evaluate the response of burley tobacco to low nitrogen. The results of this study will enable in-depth analyses of the effects of nitrogen deficiency in burley tobacco and help identify promising genes associated with nitrogen deficiency. The results may be useful in engineering burley tobacco plants that allow a reduction in the amount of chemical nitrogen fertiliser required while maintaining high crop yields.

## Results

### Leaf biomass and RNA-Seq analyses under N-deficient conditions

**Figure 1:**
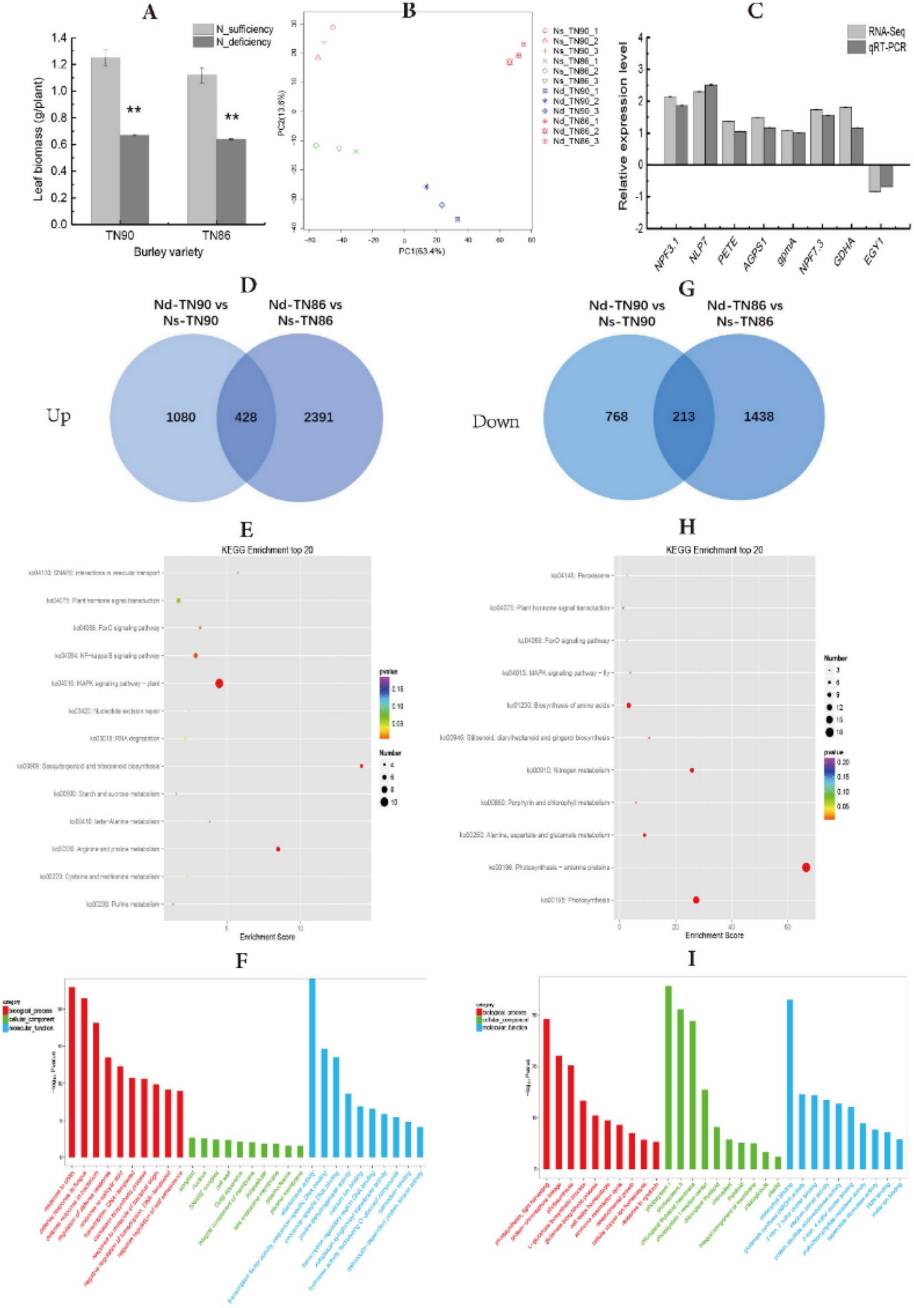
RNA-Seq analysis of burley tobacco response to nitrogen deficiency. (**A**) Leaf biomass; (**B**) PCA; (**C**) RNA-Seq results confirmed by quantitative qRT-PCR; (**D**) Venn diagram of upregulated DEGs under nitrogen-deficient conditions vs nitrogen-sufficient conditions in burley tobacco varieties TN90 and TN86; (**E**) KEGG enrichment in common upregulated DEGs in TN90 and TN86; (**F**) GO enrichment in common upregulated DEGs in TN90 and TN86; (**G**) Venn diagram of downregulated DEGs under nitrogen-deficient conditions vs nitrogen-sufficient conditions in TN90 and TN86; (**H**) KEGG enrichment in common downregulated DEGs in TN90 and TN86; (**I**) GO enrichment in common downregulated DEGs in TN90 and TN86. *Nd-TN90 *TN90 cultivated under nitrogen-deficient conditions, *Ns-TN90* TN90 cultivated under nitrogen-sufficient conditions, *Nd-TN86* TN86 cultivated under nitrogen-deficient conditions, *Ns-TN86* TN86 cultivated under nitrogen-sufficient conditions. Error bars of leaf biomass indicate standard error of the means (n = 3). Error bars of qRT-PCR indicate standard error of the means (n = 6). Symbol ** indicates a statistically significant differences between the nitrogen-deficient and nitrogen-sufficient nitrogen at *p* < 0.01.

Nitrogen deficiency significantly decreased burley tobacco leaf biomass by 52.88% (*p* < 0.01) (Fig. 1A). To evaluate the effect of nitrogen deficiency on gene expression in burley tobacco, twelve complementary DNA (cDNA) libraries (two varieties × two nitrogen application levels × three biological replicates) were analysed using RNA-Seq-based technology. Principal component analysis (PCA) results revealed there were significant differences between treatments (Fig. 1B). In total, 72.56 million reads were obtained after removing sequencing adaptors and low-quality reads. Based on the average of TN90 and TN86, 84.98% of the reads were mapped to the reference genome.

A total of 6318 differentially expressed genes (DEGs) induced by nitrogen deficiency in both TN90 and TN86 were analysed (Fig. 1). Among the DEGs, 428 upregulated genes and 213 downregulated genes were observed (Fig. 1D–G). Approximately 12.85% of the upregulated genes were involved in defence response (GO:0050832, GO:0042742, and GO:0031347; Fig. 1F) and 15.96% of the downregulated genes were associated with photosynthesis (GO:0009765 and GO:0015979; Fig. 1I). Genes annotated to gene ontology cellular component (GO-CC) were highly expressed in photosystem I (GO:0009522), photosystem II (GO:0009523), chloroplast thylakoid membrane (GO:0009535), and cytosol (GO:0005829). Genes annotated to gene ontology molecular function (GO-MF) included chlorophyll binding (GO:0016168) and glutamate synthase (NADH) activity (GO:0016040). The results indicated that genes in burley tobacco involved in photosynthesis were suppressed under conditions of reduced nitrogen application, which was consistent with the decreased accumulation of leaf biomass.

Eight DEGs were assayed by quantitative reverse-transcription polymerase chain reaction (qRT-PCR) to validate the accuracy of the RNA-Seq data (Fig. 1C). Expression patterns of the selected genes were similar in the qRT-PCR and RNA-Seq analyses, indicating the RNA-Seq results were reliable.

### Photosynthesis, carbohydrate metabolism, and carbon metabolism responses to N-deficiency

**Figure 2:**
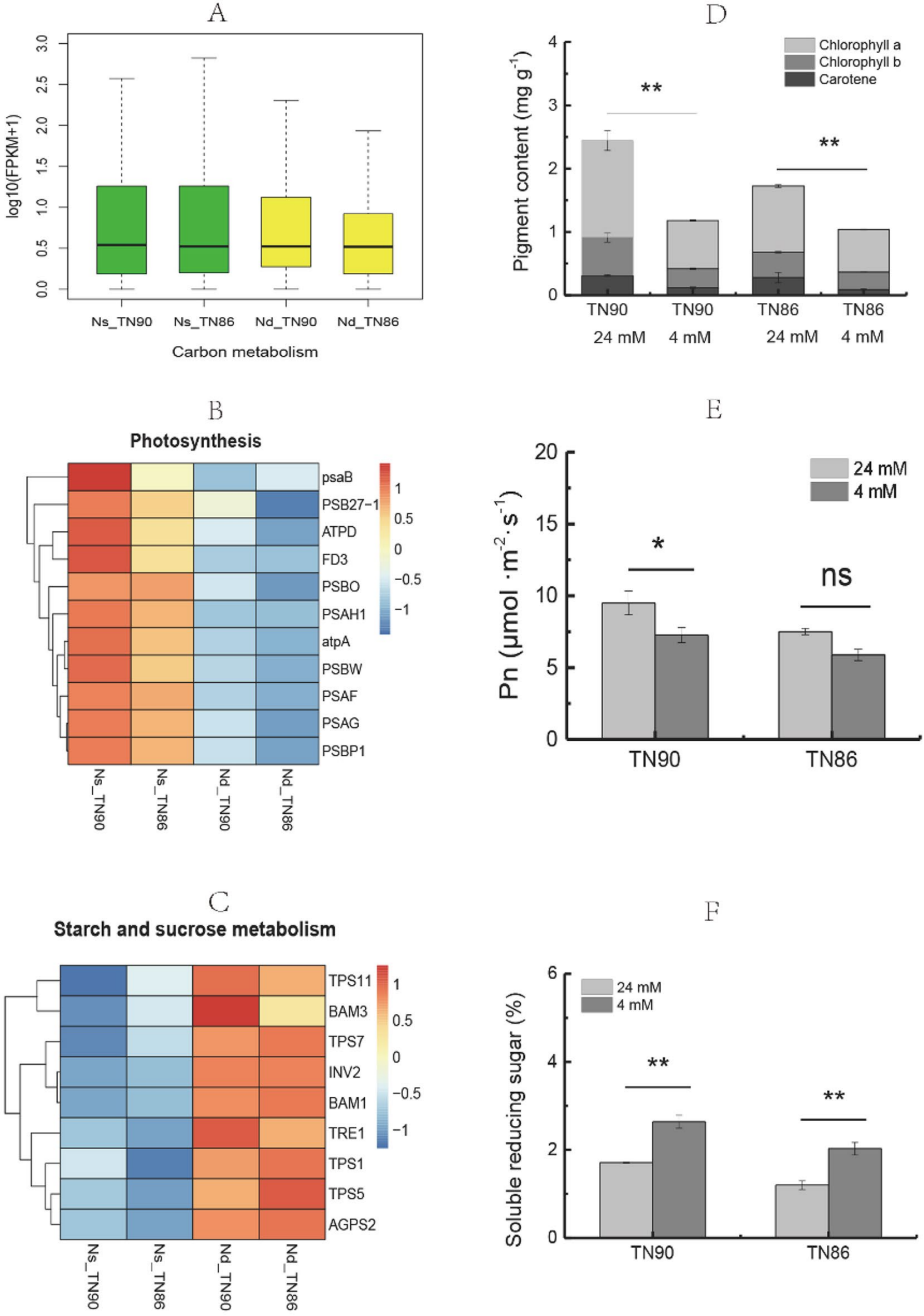
Carbon metabolism of burley tobacco under N-deficient conditions. (**A**) Primary transcripts associated with carbon metabolism; (**B**) expression pattern of genes related to photosynthesis; (**C**) expression pattern of genes related to starch and sucrose metabolism; (**D**) pigment levels; (**E**) photosynthesis rate (Pn); (**F**) soluble reducing sugar content. Symbol * and ** indicate a statistically significant differences between the nitrogen-deficient and nitrogen-sufficient conditions at *p* < 0.05 and *p* < 0.01, respectively. ns indicates no statistically significant difference between nitrogen-deficient and nitrogen-sufficient conditions at *p* < 0.05 or *p* < 0.01, respectively. *Nd-TN90* TN90 cultivated under nitrogen-deficient conditions, *Ns-TN90* TN90 cultivated under nitrogen-sufficient conditions, *Nd-TN86* TN86 cultivated under nitrogen-deficient conditions, *Ns-TN86* TN86 cultivated under nitrogen-sufficient conditions. Error bars of leaf biomass indicate standard error of the means (n = 3).

Carbon metabolism in TN90 and TN86 burley tobacco was reduced by N-deficiency (Fig. 2A–F). Among the DEGs downregulated by N-deficient conditions, 11 were associated with the photosynthesis pathway, indicating photosynthesis in burley tobacco would be decreased by reducing the application of nitrogen. Pigment concentration and Pn were decreased in TN90 and TN86 under N-deficient conditions (pigment: 51.71% and 39.85%, *p* < 0.01, respectively; Pn: 23.53%, *p* < 0.05 and 21.55%, *p* > 0.05, respectively). Among the DEGs upregulated under N-deficient conditions, 11 were associated with starch and sucrose metabolism pathways. Furthermore, soluble reducing sugar content in TN90 and TN86 was significantly increased by 54.28% and 69.17%, (*p* < 0.01), respectively, indicating carbohydrate biosynthesis in burley tobacco was increased in response to reducing the application of nitrogen.

### Nitrogen metabolism response to N-deficiency

**Figure 3:**
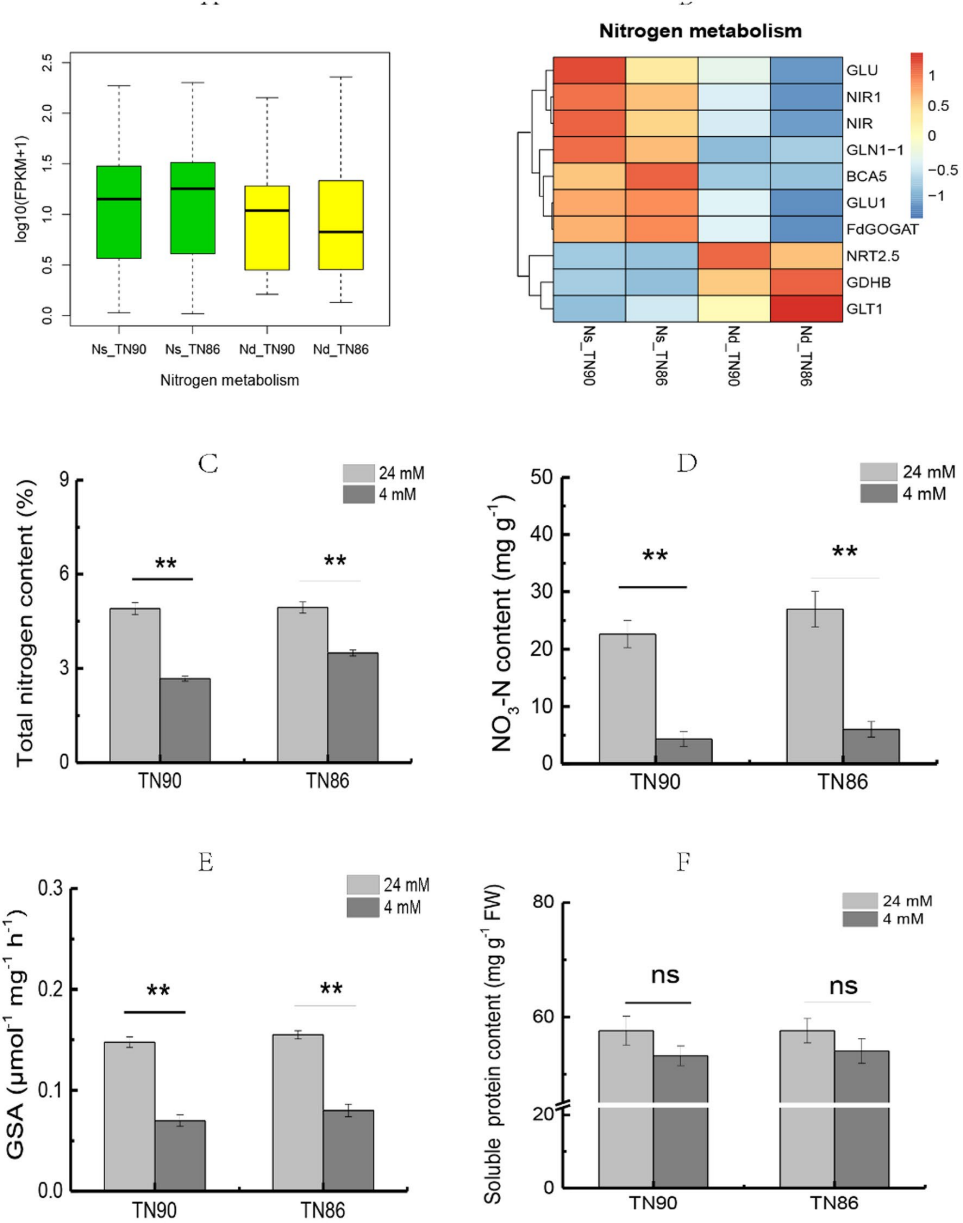
Nitrogen metabolism of burley tobacco under N-deficiency conditions. (**A**) Primary transcripts associated with nitrogen metabolism; (**B**) expression pattern of genes related to nitrogen metabolism; (**C**) total nitrogen content; (**D**) NO_3_-N content; (**E**) GSA activity; (**F**) soluble protein content. Symbol ** indicate a statistically significant differences between nitrogen-deficient and nitrogen-sufficient conditions at *p* < 0.01. ns indicates no statistically significant difference between nitrogen-deficient and nitrogen-sufficient conditions at *p* < 0.05 and *p* < 0.01, respectively. Error bars of leaf biomass indicate standard error of the means (n = 3).

Nitrogen metabolism in burley tobacco was suppressed under N-deficient conditions (Fig. 3A–F). DEGs downregulated by reducing the application of nitrogen included 10 that were associated with nitrogen assimilation. In contrast, genes associated with nitrate response and nitrate transporters were upregulated. Total nitrogen content, NO_3_-N content, and glutamine synthetase (GSA) activity under N-deficient conditions were significantly decreased by 45.37%, 80.85%, and 52.60%, respectively, in TN90 and 29.53%, 77.72%, and 48.39%, respectively, in TN86 (*p* < 0.01). Soluble protein content was decreased by 7.59% in TN90 and 6.15% in TN86, indicating nitrogen assimilation was suppressed and nitrate transport was enhanced when the application of nitrogen was reduced.

### Root development and chemical content in response to N-deficiency

**Figure 4:**
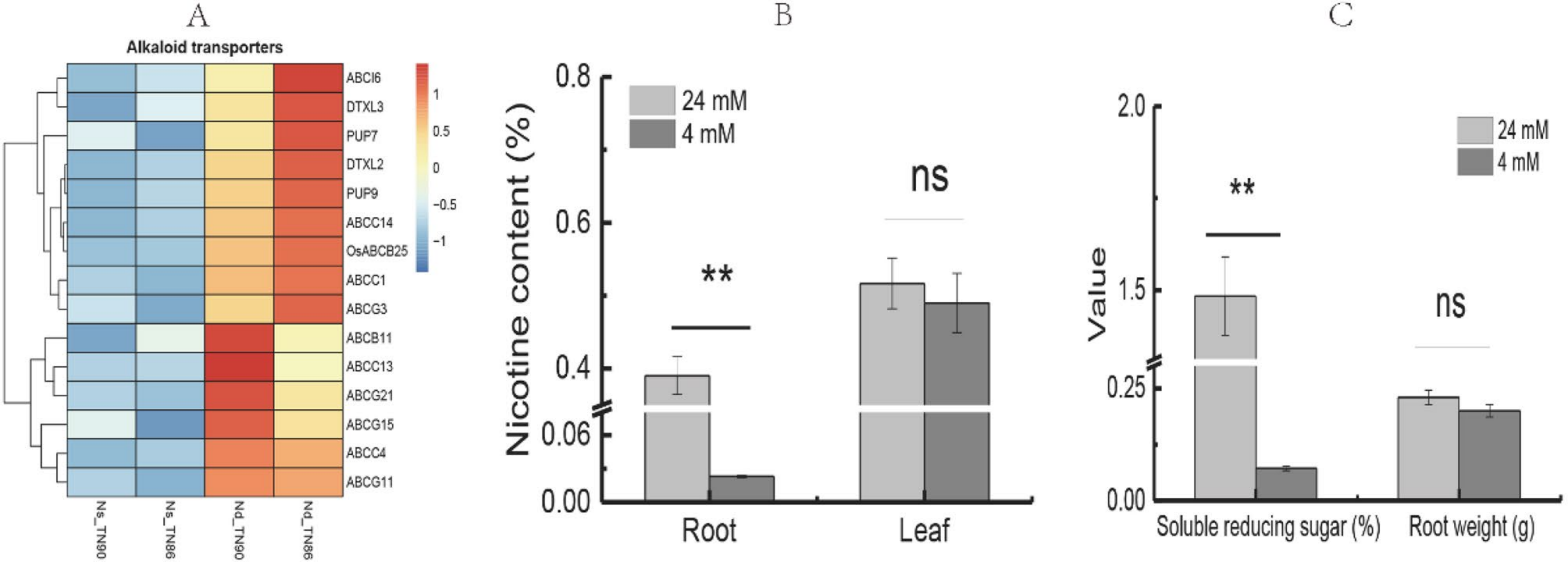
Effects of N-deficient conditions on alkaloid transporters, nicotine, soluble reducing sugar, and weight of tobacco roots. (**A**) Expression pattern of genes related to alkaloid transports; (**B**) nicotine content; (**C**) soluble reducing sugar content and root weight. Symbol ** indicates a statistically significant differences between nitrogen-deficient and nitrogen-sufficient conditions at *p* < 0.01. ns indicates no statistically significant difference between nitrogen-deficient and nitrogen-sufficient conditions at *p* < 0.05 and *p* < 0.01, respectively. Error bars of leaf biomass indicate standard error of the means (n = 3).

Nicotine levels in the roots and leaves of burley tobacco were decreased under N-deficient conditions, especially in the roots (Fig. 4A–C). Soluble reducing sugar content and root weight were significantly decreased by reducing the application of nitrogen (*p* < 0.01). Root length, root surface, root diameter, and root volume in TN86 under N-deficient conditions were decreased by 16.63%, 52.82%, 42.22%, and 73.56%, respectively (Table 1), indicating that root morphology was suppressed and energy production was insufficient, which would lead to decreased nicotine biosynthesis. Among the DEGs upregulated by N-deficiency, 15 were annotated to alkaloid transporters. The alkaloid transport-related genes were mainly enriched in ABC transporters, multidrug and toxic compound extrusion (MATE), and purine permease (PUP) families.

**Table 1:** Effects of nitrogen-sufficient and nitrogen-deficient conditions on burley tobacco root morphology.

Treatment	Length (cm)	Surface area (cm^2^)	Average diameter (mm)	Root volume (cm^3^)	Tips
Ns	387.68 ± 44.49a	57.59 ± 6.61a	0.45 ± 0.05a	0.69 ± 0.08a	443.61 ± 50.91a
Nd	323.19 ± 28.34a	27.17 ± 2.38b	0.26 ± 0.02b	0.18 ± 0.02b	444.26 ± 38.95a

Values followed by lowercase letters in the same column are significant by different treatments at a probability level of 0.05. *Ns* nitrogen-sufficient conditions, *Nd* nitrogen-deficient conditions.

### Signalling response to N-deficiency

**Figure 5:**
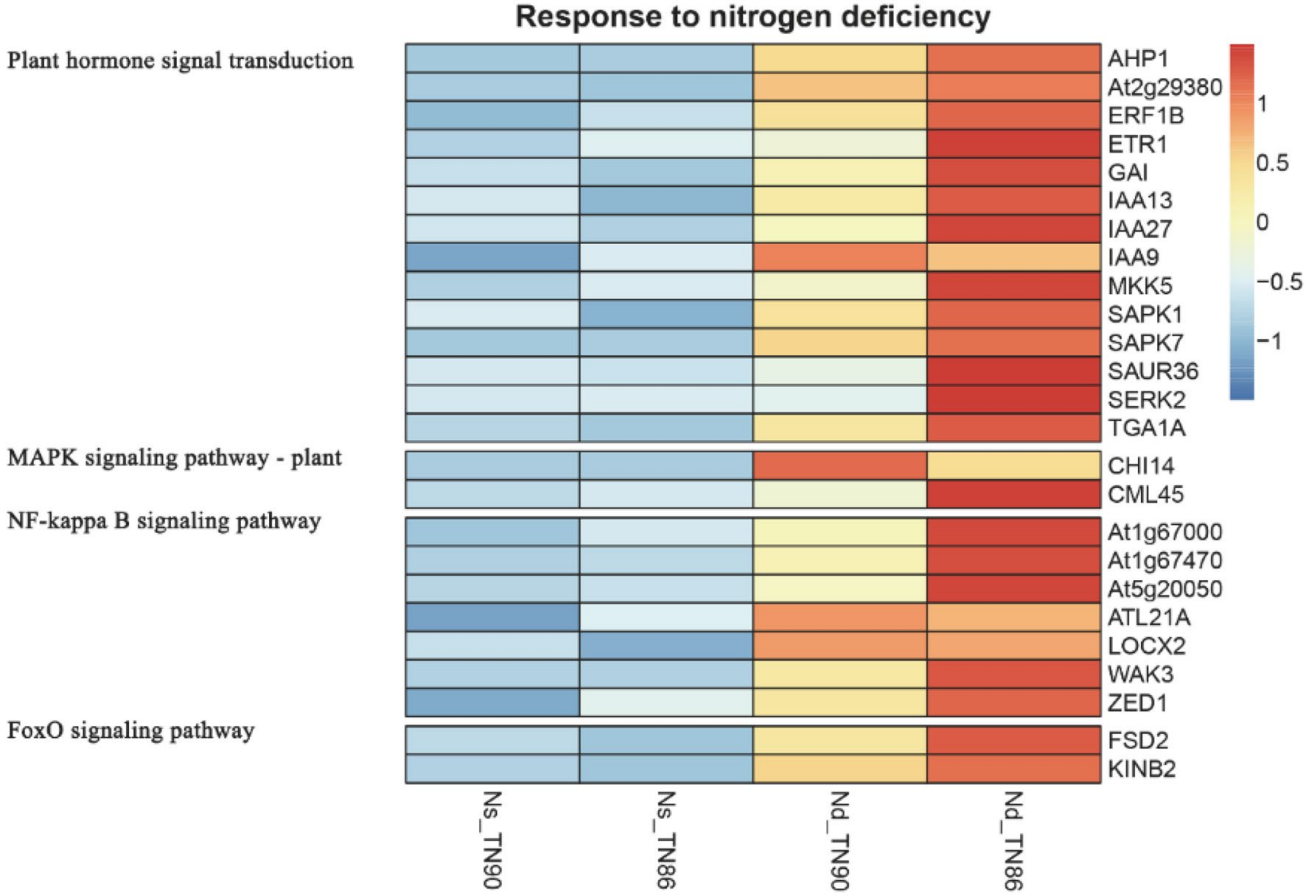
Expression pattern of genes related to nitrogen deficiency responses. *Nd-TN90* TN90 cultivated under nitrogen-deficient conditions, *Ns-TN90* TN90 cultivated under nitrogen-sufficient conditions, *Nd-TN86* TN86 cultivated under nitrogen-deficient conditions, *Ns-TN86* TN86 cultivated under nitrogen-sufficient conditions.

Clustering analysis was performed to evaluate the differentially expressed genes involved in signalling in the two burley tobacco varieties under N-deficient conditions and the results are demonstrated in the heatmap shown in Fig. 5. A total of 25 DEGs were annotated to plant hormone signal transduction with the MAPK signalling pathway, NF-kappa B signalling pathway, and FoxO signalling pathway being upregulated by N-deficiency. Moreover, there were more differentially expressed genes in TN86 compared to those in TN90. Expression of *At2g29380*, *ATL21A*, and *LOCX2* were upregulated in both TN90 and TN86 under N-deficient conditions, indicating N-deficient conditions promoted the expression of genes associated with serine-threonine protein phosphatase and putative ring-H2 finger protein.

## Discussion

To gain the same leaf biomass for burley tobacco as that for other tobacco types requires almost a six-fold higher application of nitrogen^21^, which demonstrates that nitrogen utilization efficiency in burley tobacco is low. To explore the effects of nitrogen deficiency on burley tobacco, the transcriptome, physiology, and biochemistry of two burley varieties were evaluated under N-sufficient and N-deficient conditions. The carbohydrate and nitrogenous compound content, nicotine, nitrogen, and carbon metabolism, alkaloid translation, response to nitrogen deficiency, and root architecture were all analysed.

In our study, the leaf biomass of burley tobacco plants was significantly decreased by nitrogen deficiency (*p* < 0.01). Nitrogen is an essential factor that can influence the development and biomass of plants^22,23^. We found that total nitrogen and nitrate contents were significantly decreased in the two burley varieties under N-deficient conditions (*p* < 0.01), indicating the accumulation of total nitrogen and available nitrogen in burley tobacco was not high under N-deficient conditions. Burley tobacco is characterised by high nitrogenous content and low carbohydrate content in its leaves^24^. In our previous work, soluble protein content was much lower in burley tobacco compared with that in other tobacco types^24^. However, nitrogen metabolism and the amount of nitrogen in TN90 and TN86 were similar under the same nitrogen conditions. In the current study, soluble protein content was reduced in response to nitrogen deficiency, but not significantly. This may be a natural characteristic of burley tobacco^24^. *NTR2.5* (a high-affinity nitrogen translator), *GDHB* (the gene encoding glutamate dehydrogenase B), and *GLT1* (a gene involved in the ammonium assimilation pathway) are related to nitrogen translation and nitrogen assimilation^25–28^. The expression of *NRT2.5*, *GDHB*, and *GLT1* was increased in the current study in response to nitrogen deficiency, indicating the ability of nitrogen translation and assimilation in burley tobacco leaves may be enhanced under nitrogen deficiency conditions.

Carbon metabolism and nitrogen metabolism in plants are very closely related^29^. Nitrogen metabolism supplies amino acids, enzyme proteins, and photosynthetic pigments to carbon metabolism, while carbon metabolism supplies carbon atoms and energy to nitrogen metabolism^29,30^. Expression of genes related to carbon metabolism and photosynthesis decreased under nitrogen-deficient conditions. The Pn and total sugar content (not shown) were significantly decreased in TN90 under N-deficiency conditions (*p* < 0.05), but not in TN86. This may have been due to the lower pigment levels in TN86 compared to that in TN90. In general, low nitrogen can accelerate senescence by significantly reducing pigment content, Pn, and total sugar content in plant leaves^31–33^, the Pn of TN86 did not significantly decrease in our study, indicating carbon metabolism in TN86 may be complex^34,35^. In our previous work, an exogenous carbon source sprayed onto the leaf of burley tobacco enhanced nitrogen translation and assimilation^21^. The amount of soluble reducing sugar supplying energy to nitrogen translation and assimilation in the two burley tobacco plant varieties was significantly decreased under N-deficient conditions. This indicated that no excess soluble reducing sugar was available to accumulate, which had a negative effect on leaf biomass formation and accrual. Expression of trehalose phosphatase/synthase 11 (*TPS11*)*,* trehalose-phosphatase/synthase 7 (*TPS7*)*,* beta-fructofuranosidase (*INV2*)*,* the leucine-rich receptor-like protein kinase family protein *β-amylase 1* (*BAM1*)*,* and ADP-glucose pyrophosphorylase small subunit CagpS2 (*AGPS2*) were decreased, which may have been due to decreased soluble reducing sugar content.

Nicotine biosynthesis in roots is an important parameter for evaluating the quality of tobacco. Root growth and weight are significantly suppressed under low nitrogen conditions^36^. Nicotine levels were decreased in burley tobacco under N-deficient conditions, especially in the roots, which may have been a result of low soluble reducing sugar content or smaller surface area, average diameter, and root volume of the roots. In contrast, the expression in leaves of genes involved in alkaloid transport was increased under N-deficient conditions, which reduced the impact of decreasing nicotine in the aboveground parts of burley tobacco plants.

In conclusion, nitrogen deficiency significantly affected carbon and nitrogen metabolism in burley tobacco and expression of genes related to carbon and nitrogen metabolism were decreased. Leaf biomass and total nitrogen, nitrate, soluble reducing sugar, and nicotine content were all significantly decreased under N-deficient conditions (*p* < 0.01). Carbon metabolism was more complex in TN86 than that in TN90. Finally, nitrogen deficiency negatively affected the development of root morphology, but was positive for nitrogen, nitrate, and nicotine transporters.

## Material and methods

### Plant material and treatments

Two varieties of burley tobacco (TN86 and TN90) and four treatment groups were used in this study. The treatment groups were as follows: (1) TN90 with 24 mmol/L nitrogen; (2) TN86 with 24 mmol/L nitrogen; (3) TN90 with 4 mmol/L nitrogen; and (4) TN86 with 4 mmol/L nitrogen. A nitrogen concentration of 24 mmol/L was considered N-sufficient and a concentration of 4 mmol/L was considered N-deficient. Plants for the experiments were prepared on substrate culture in a greenhouse maintained at a temperature of 23 ± 2 °C. Seeds were sterilised with 2% (v/v) sodium hypochlorite and then sown in a floating system. After 40 days, the seedlings were transplanted into 25 cm diameter × 30 cm depth plastic pots and placed onto trays. Specimens were screened and selected from three independent test plots. The composition of the nutrient solution was determined using the method described by Hoagland^21^. The ratio of NO_3_^-^ to NH_4_^+^ was 1:3 in both the N-sufficient and N-deficient treatments. The nutrient solution decanted onto the trays was replaced daily.

### Biomass accumulation and root morphology assessment

The plant specimens were dissected into leaves, stems, and roots and cleaned with distilled water. Fresh leaves were fixed for 20 min at 105 °C and then dried for 48 h at 60 °C. Roots were kept moist with deionised water at 4 °C and then scanned using an Epson Expression 10000XL digital scanner (Epson Electronics Inc., San Jose, CA, USA). The images were analysed using WinRHIZO 2012b root analysis software (Regent Instrument Inc. Canada) to quantify the main root parameters. Root length, root superficial area, average diameter, root volume, and root tip were calculated using the data as previously described^37^.

### Measurement of GSA activity, Pn, and nitrate, pigment, total nitrogen, and soluble reducing sugar content

Fresh lamina tissues without veins were cut into 2 mm × 5 mm pieces. GSA activity was determined according to the method described by O’Neal and Joy^38^. Nitrate content was determined according to the method described by Cataldo^39^. Pigment content was determined using 95% ethanol as previously described^40^. Pn was measured at 9:00–11:00 a.m. using a 6400XT portable photosynthesis system (LI-COR Biotechnology, Lincoln, NE, USA) as described by Liu^41^. Total nitrogen, nicotine, and soluble reducing sugar content were determined according to the modified Chinese Tobacco industry standard (YC/T 161, 159-2002)^24^.

### RNA extraction and sequencing

RNA was extracted from each sample using the methods described previously^21^. Samples with an RNA integrity number (RIN) ≥ 7 were subjected to subsequent analysis. The sequencing format was 125 paired-end or 150 paired-end. After removal of low-quality data^42^, the clean reads with Q20 percentage of 94.07% were mapped to the *Populus trichocarpa* reference genome using bowtie2 or Tophat (http://tophat.cbcb.umd.edu/)^43,44^.

### RNA-Seq, GO, and KEGG pathway enrichment analyses of DEGs

Transcript profiles of the RNA-Seq data were analysed by calculating the read fragments per kilobase per million mapped reads (FPKM). The FPKM value of each gene was calculated using cufflinks and the read counts of each gene obtained using htseq-count^45,46^. DEGs were identified using the DESeq (2012 update) function to estimate size factors and nbinomTest to test for differences between base means^47^. A *p* value < 0.05 and fold change > 2 or fold change < 0.5 was set as the threshold for significantly differential expression. Gene function was annotated based on data from databases, including the National Center for Biotechnology Information (NCBI) non-redundant protein sequences NR database (ftp://ftp.ncbi.nlm.nih.gov/blast/db/), the KOG database of Clusters of Orthologous Groups of proteins (http://www.ncbi.nlm.nih.gov/COG/)^48^, the manually annotated and reviewed protein sequence database Swiss-Prot (http://web.expasy.org/docs/swiss-prot_guideline.html), the Kyoto Encyclopedia of Genes and Genomes (KEGG) Ortholog (KO) database (http://www.genome.jp/kegg/), and Gene Ontology (GO; http://www.geneontology.org)^49–51^ (https://www.ncbi.nlm.nih.gov/). GO enrichment analysis (https://bioconductor.org/packages/release/data/annotation/html/GO.db.html) and KEGG pathway enrichment analysis (https://bioconductor.org/packages/release/data/annotation/html/KEGG.db.html) of DEGs were performed based on hypergeometric distribution using R. PCA and Heatmap analysis of DEGs were performed using R, version 3.4.1 (https ://www.r-proje ct.org/; Lucent Technologies, Murray Hill, NJ, USA)^52^. Box plots were plotted using Bioconductor affycomp (https://bioconductor.org/packages/release/bioc/html/affycomp.html) according to the methods of Jin^53^.

### Validation of RNA-Seq using qRT-PCR analysis

Eight genes obtained in the RNA-Seq results were randomly selected and validated by qRT-PCR. Quantification was performed using a two-step reaction process of reverse transcription (RT) and PCR amplification. Reactions were incubated in a 384-well optical plate (Roche, Basel, Switzerland) at 95 °C for 5 min, followed by 40 cycles of 95 °C for 10 s and 60 °C for 30 s. The primer sequences were designed in house based on the mRNA sequences obtained from the NCBI database and synthesised by Generay Biotech (Shanghai, China). The mRNA expression levels were normalised and calculated using the 2^−ΔΔCt^ method^54^.

### Statistical analysis

The figures were prepared using Origin Pro 9.0 software (Origin Lab Corporation, Northampton, MA, USA). Variance analysis was performed using IBM SPSS version 20.0 software (IBM, Palo Alto, CA, USA) at a level of 0.05 with the least significant difference (LSD) multiple range test (*p* < 0.05).

### Ethics statement

The tobacco varieties were supplied by China National Infrastructure for Crop Germplasm Resources (Tobacco, Qingdao), including TN90 and TN86 varieties. Experimental research and field studies on plants in this work, including the collection of plant material, comply with relevant institutional, national, and international guidelines and legislation.

## Supplementary Information

Supplementary Information 1.
